# Ethyl 1-phenyl­sulfonyl-1*H*-indole-2-carboxyl­ate

**DOI:** 10.1107/S1600536811035392

**Published:** 2011-09-03

**Authors:** C. Ramathilagam, V. Saravanan, A. K. Mohanakrishnan, P. R. Umarani, V. Manivannan

**Affiliations:** a Department of Physics, AMET University, Kanathur, Chennai 603 112, India; b Department of Organic Chemistry, University of Madras, Guindy Campus, Chennai 600 025, India; c Department of Physics, Presidency College (Autonomous), Chennai 600 005, India; d Department of Research and Development, PRIST University, Vallam, Thanjavur 613 403, Tamil Nadu, India

## Abstract

In the title compound, C_17_H_15_NO_4_S, the six-membered ring of the indole unit makes a dihedral angle of 72.40 (5)° with the phenyl ring. The mol­ecular structure features a short C—H⋯O contact.

## Related literature

For the biological activity of Indole derivatives, see: Joshi & Chand (1982 ▶); Pomarnacka & Kozlarska-Kedra (2003 ▶); For a related structure, see: Chakkaravarthi *et al.* (2010 ▶).
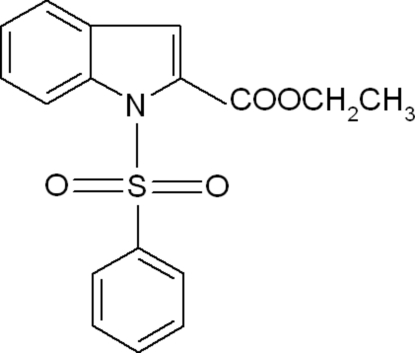


## Experimental

### 

#### Crystal data

C_17_H_15_NO_4_S
*M* *_r_* = 329.36
Monoclinic, 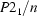

*a* = 10.6936 (6) Å
*b* = 7.5331 (4) Å
*c* = 19.5654 (12) Å
β = 96.647 (2)°
*V* = 1565.52 (15) Å^3^
*Z* = 4
Mo *K*α radiation
μ = 0.23 mm^−1^
*T* = 295 K
0.22 × 0.20 × 0.18 mm

#### Data collection

Bruker Kappa APEXII CCD diffractometer
Absorption correction: multi-scan (*SADABS*; Sheldrick, 1996 ▶) *T*_min_ = 0.952, *T*_max_ = 0.960
35524 measured reflections
4353 independent reflections
3245 reflections with *I* > 2σ(*I*)
*R*_int_ = 0.041

#### Refinement

*R*[*F* ^2^ > 2σ(*F* ^2^)] = 0.042
*wR*(*F* ^2^) = 0.129
*S* = 1.02
4353 reflections
209 parameters
H-atom parameters constrained
Δρ_max_ = 0.33 e Å^−3^
Δρ_min_ = −0.23 e Å^−3^

### 

Data collection: *APEX2* (Bruker, 2004 ▶); cell refinement: *SAINT* (Bruker, 2004 ▶); data reduction: *SAINT*; program(s) used to solve structure: *SHELXS97* (Sheldrick, 2008 ▶); program(s) used to refine structure: *SHELXL97* (Sheldrick, 2008 ▶); molecular graphics: *PLATON* (Spek, 2009 ▶); software used to prepare material for publication: *SHELXL97* .

## Supplementary Material

Crystal structure: contains datablock(s) global, I. DOI: 10.1107/S1600536811035392/bt5624sup1.cif

Structure factors: contains datablock(s) I. DOI: 10.1107/S1600536811035392/bt5624Isup2.hkl

Supplementary material file. DOI: 10.1107/S1600536811035392/bt5624Isup3.cml

Additional supplementary materials:  crystallographic information; 3D view; checkCIF report

## Figures and Tables

**Table 1:** Hydrogen-bond geometry (Å, °)

*D*—H⋯*A*	*D*—H	H⋯*A*	*D*⋯*A*	*D*—H⋯*A*
C2—H2⋯O1	0.93	2.29	2.839 (2)	117

**Fig. 1.**
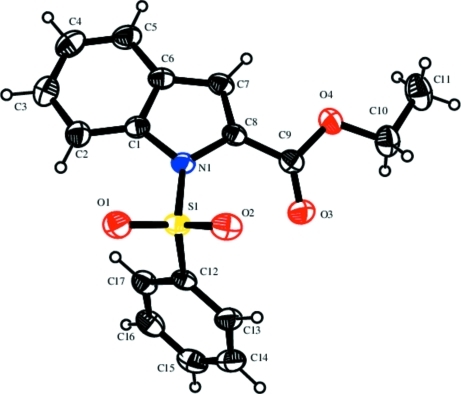
The molecular structure of the title compound, with atom labels and 30% probability displacement ellipsoids for non-H atoms.

## supplementary crystallographic information

### Comment

Compounds containing the indole moiety exhibit antibacterial and fungicidal activities (Joshi & Chand, 1982). In addition, indole derivatives are also known to exhibit anticancer and anti-HIV (Pomarnacka & Kozlarska-Kedra, 2003) activities.

The geometric parameters of the title molecule (Fig. 1) agree well with reported similar structures (Chakkaravarthi *et al.*, 2010) The dihedral angle between the six membered ring of the indole moiety and the benzene ring is 72.40 (5)°. The sum of bond angles around N1 [359.03 (9) °] indicates the *sp^2^* hybridization state. The molecular structure is stabilized by weak intramolecular C—H···O interaction.

### Experimental

Ethyl-indole-2- carboxylate (1 g,5.29 mmol) was dissolved in distilled benzene (20 ml). To this benzenesulfonyl chloride (0.82 ml,5.82 mmol) and 60% NaOH (2.1 g in3.52 ml) were added along with tetra butyl ammonium hydrogen sulfate (1.0 g). This two phase system was stirred at room temperature for 2 h. It was then diluted with water (50 ml)and the organic layer was separated. The aqueous layer was extracted with benzene (2x20 ml)and the combined organic extracts were dried(Na_2_SO_4_). The solvent was removed completely and the crude product was recrystallized from methanol afforded ethyl- 1-phenylsulfonyl-indole 2-carboxylate (m.p 395–397 K).

### Refinement

H atoms were positioned geometrically and refined using riding model with C—H = 0.93Å and *U*_iso_(H) = 1.2Ueq(C) for aromatic C—H, C—H = 0.97Å and *U*_iso_(H) = 1.2Ueq(C) for CH2, C—H = 0.96Å and *U*_iso_(H) = 1.5Ueq(C) for CH_3_.

### Crystal data

**Table:**

C_17_H_15_NO_4_S	*F*(000) = 688
*M**_r_* = 329.36	*D*_x_ = 1.397 Mg m^−^^3^
Monoclinic, *P*2_1_/*n*	Mo *K*α radiation, λ = 0.71073 Å
Hall symbol: -P 2Yn	Cell parameters from 4353 reflections
*a* = 10.6936 (6) Å	θ = 2.1–29.5°
*b* = 7.5331 (4) Å	µ = 0.23 mm^−^^1^
*c* = 19.5654 (12) Å	*T* = 295 K
β = 96.647 (2)°	Block, colourless
*V* = 1565.52 (15) Å^3^	0.22 × 0.20 × 0.18 mm
*Z* = 4	

### Data collection

**Table:**

Bruker Kappa APEXII CCD diffractometer	4353 independent reflections
Radiation source: fine-focus sealed tube	3245 reflections with *I* > 2σ(*I*)
graphite	*R*_int_ = 0.041
Detector resolution: 0 pixels mm^-1^	θ_max_ = 29.5°, θ_min_ = 2.1°
ω and φ scans	*h* = −14→14
Absorption correction: multi-scan (*SADABS*; Sheldrick, 1996)	*k* = −10→10
*T*_min_ = 0.952, *T*_max_ = 0.960	*l* = −26→27
35524 measured reflections	

### Refinement

**Table:**

Refinement on *F*^2^	Primary atom site location: structure-invariant direct methods
Least-squares matrix: full	Secondary atom site location: difference Fourier map
*R*[*F*^2^ > 2σ(*F*^2^)] = 0.042	Hydrogen site location: inferred from neighbouring sites
*wR*(*F*^2^) = 0.129	H-atom parameters constrained
*S* = 1.02	*w* = 1/[σ^2^(*F*_o_^2^) + (0.0661*P*)^2^ + 0.3737*P*] where *P* = (*F*_o_^2^ + 2*F*_c_^2^)/3
4353 reflections	(Δ/σ)_max_ = 0.001
209 parameters	Δρ_max_ = 0.33 e Å^−^^3^
0 restraints	Δρ_min_ = −0.23 e Å^−^^3^

### Special details

**Table:**

Geometry. All e.s.d.'s (except the e.s.d. in the dihedral angle between two l.s. planes) are estimated using the full covariance matrix. The cell e.s.d.'s are taken into account individually in the estimation of e.s.d.'s in distances, angles and torsion angles; correlations between e.s.d.'s in cell parameters are only used when they are defined by crystal symmetry. An approximate (isotropic) treatment of cell e.s.d.'s is used for estimating e.s.d.'s involving l.s. planes.
Refinement. Refinement of *F*^2^ against ALL reflections. The weighted *R*-factor *wR* and goodness of fit *S* are based on *F*^2^, conventional *R*-factors *R* are based on *F*, with *F* set to zero for negative *F*^2^. The threshold expression of *F*^2^ > σ(*F*^2^) is used only for calculating *R*-factors(gt) *etc*. and is not relevant to the choice of reflections for refinement. *R*-factors based on *F*^2^ are statistically about twice as large as those based on *F*, and *R*- factors based on ALL data will be even larger.

### Fractional atomic coordinates and isotropic or equivalent isotropic displacement parameters (Å^2^)

**Table:**

	*x*	*y*	*z*	*U*_iso_*/*U*_eq_	
C1	0.75477 (14)	0.1614 (2)	0.14296 (8)	0.0459 (3)	
C2	0.76555 (18)	0.1553 (3)	0.07299 (10)	0.0595 (4)	
H2	0.6951	0.1613	0.0404	0.071*	
C3	0.8851 (2)	0.1399 (3)	0.05402 (12)	0.0707 (5)	
H3	0.8950	0.1358	0.0075	0.085*	
C4	0.99095 (19)	0.1305 (3)	0.10177 (13)	0.0746 (6)	
H4	1.0700	0.1195	0.0868	0.090*	
C5	0.98078 (17)	0.1371 (3)	0.16998 (12)	0.0667 (5)	
H5	1.0523	0.1320	0.2019	0.080*	
C6	0.86132 (15)	0.1518 (2)	0.19186 (9)	0.0500 (4)	
C7	0.82011 (15)	0.1562 (2)	0.25786 (9)	0.0517 (4)	
H7	0.8717	0.1525	0.2995	0.062*	
C8	0.69340 (14)	0.1666 (2)	0.25026 (8)	0.0444 (3)	
C9	0.61219 (15)	0.1458 (2)	0.30554 (9)	0.0479 (4)	
C10	0.6183 (2)	0.1412 (3)	0.42624 (10)	0.0699 (5)	
H10A	0.5795	0.0247	0.4225	0.084*	
H10B	0.5535	0.2287	0.4314	0.084*	
C11	0.7173 (3)	0.1476 (4)	0.48626 (12)	0.0924 (8)	
H11A	0.7850	0.0694	0.4783	0.139*	
H11B	0.6821	0.1109	0.5269	0.139*	
H11C	0.7487	0.2667	0.4922	0.139*	
C12	0.42232 (13)	0.0165 (2)	0.14176 (8)	0.0443 (3)	
C13	0.31930 (15)	−0.0030 (3)	0.17714 (10)	0.0562 (4)	
H13	0.2935	0.0894	0.2038	0.067*	
C14	0.25463 (18)	−0.1627 (3)	0.17237 (11)	0.0653 (5)	
H14	0.1838	−0.1774	0.1952	0.078*	
C15	0.2945 (2)	−0.2984 (3)	0.13425 (11)	0.0682 (5)	
H15	0.2515	−0.4060	0.1320	0.082*	
C16	0.3978 (2)	−0.2779 (3)	0.09909 (11)	0.0661 (5)	
H16	0.4242	−0.3715	0.0733	0.079*	
C17	0.46224 (17)	−0.1190 (2)	0.10196 (9)	0.0558 (4)	
H17	0.5311	−0.1034	0.0776	0.067*	
N1	0.64889 (11)	0.17160 (18)	0.17945 (7)	0.0445 (3)	
O1	0.51424 (12)	0.27385 (18)	0.07617 (7)	0.0626 (3)	
O2	0.44931 (11)	0.33875 (16)	0.18971 (7)	0.0589 (3)	
O3	0.50501 (12)	0.09756 (19)	0.29867 (7)	0.0646 (3)	
O4	0.67930 (12)	0.17905 (19)	0.36602 (6)	0.0611 (3)	
S1	0.50234 (3)	0.22047 (5)	0.14475 (2)	0.04621 (13)	

### Atomic displacement parameters (Å^2^)

**Table:**

	*U*^11^	*U*^22^	*U*^33^	*U*^12^	*U*^13^	*U*^23^
C1	0.0410 (8)	0.0423 (7)	0.0540 (9)	0.0003 (6)	0.0037 (6)	0.0018 (6)
C2	0.0572 (10)	0.0664 (11)	0.0551 (10)	−0.0002 (8)	0.0071 (8)	0.0046 (8)
C3	0.0731 (13)	0.0750 (13)	0.0678 (12)	0.0045 (10)	0.0247 (10)	0.0025 (10)
C4	0.0519 (11)	0.0801 (14)	0.0961 (17)	0.0050 (10)	0.0263 (11)	0.0025 (12)
C5	0.0409 (9)	0.0747 (13)	0.0846 (14)	0.0047 (8)	0.0071 (9)	−0.0044 (10)
C6	0.0391 (7)	0.0463 (8)	0.0633 (10)	0.0011 (6)	0.0004 (7)	−0.0017 (7)
C7	0.0412 (8)	0.0554 (9)	0.0554 (9)	0.0053 (7)	−0.0068 (7)	−0.0053 (7)
C8	0.0407 (7)	0.0433 (7)	0.0472 (8)	0.0035 (6)	−0.0035 (6)	−0.0010 (6)
C9	0.0459 (8)	0.0452 (8)	0.0510 (9)	0.0076 (6)	−0.0011 (7)	0.0006 (6)
C10	0.0802 (14)	0.0781 (13)	0.0521 (11)	0.0125 (11)	0.0106 (9)	0.0039 (9)
C11	0.116 (2)	0.1060 (19)	0.0524 (12)	0.0233 (16)	−0.0021 (12)	0.0063 (12)
C12	0.0362 (7)	0.0461 (7)	0.0474 (8)	0.0036 (6)	−0.0083 (6)	0.0005 (6)
C13	0.0407 (8)	0.0596 (10)	0.0672 (11)	0.0006 (7)	0.0012 (7)	−0.0057 (8)
C14	0.0487 (10)	0.0690 (11)	0.0761 (13)	−0.0103 (8)	−0.0017 (9)	0.0078 (10)
C15	0.0701 (12)	0.0534 (10)	0.0747 (13)	−0.0123 (9)	−0.0188 (10)	0.0068 (9)
C16	0.0773 (13)	0.0498 (9)	0.0666 (12)	0.0052 (9)	−0.0111 (10)	−0.0081 (8)
C17	0.0544 (9)	0.0557 (9)	0.0551 (10)	0.0061 (7)	−0.0027 (7)	−0.0051 (7)
N1	0.0352 (6)	0.0516 (7)	0.0452 (7)	0.0011 (5)	−0.0015 (5)	0.0022 (5)
O1	0.0546 (7)	0.0708 (8)	0.0595 (8)	0.0042 (6)	−0.0051 (6)	0.0204 (6)
O2	0.0503 (7)	0.0461 (6)	0.0784 (9)	0.0119 (5)	−0.0001 (6)	−0.0040 (6)
O3	0.0481 (7)	0.0822 (9)	0.0629 (8)	−0.0033 (6)	0.0042 (6)	0.0031 (7)
O4	0.0590 (7)	0.0773 (8)	0.0458 (7)	0.0003 (6)	0.0003 (5)	−0.0031 (6)
S1	0.0382 (2)	0.0439 (2)	0.0542 (2)	0.00539 (14)	−0.00468 (15)	0.00506 (16)

### Geometric parameters (Å, °)

**Table:**

C1—C2	1.388 (2)	C10—H10A	0.9700
C1—C6	1.402 (2)	C10—H10B	0.9700
C1—N1	1.409 (2)	C11—H11A	0.9600
C2—C3	1.376 (3)	C11—H11B	0.9600
C2—H2	0.9300	C11—H11C	0.9600
C3—C4	1.384 (3)	C12—C13	1.375 (2)
C3—H3	0.9300	C12—C17	1.381 (2)
C4—C5	1.352 (3)	C12—S1	1.7567 (16)
C4—H4	0.9300	C13—C14	1.386 (3)
C5—C6	1.398 (2)	C13—H13	0.9300
C5—H5	0.9300	C14—C15	1.363 (3)
C6—C7	1.412 (3)	C14—H14	0.9300
C7—C8	1.348 (2)	C15—C16	1.376 (3)
C7—H7	0.9300	C15—H15	0.9300
C8—N1	1.412 (2)	C16—C17	1.379 (3)
C8—C9	1.472 (2)	C16—H16	0.9300
C9—O3	1.195 (2)	C17—H17	0.9300
C9—O4	1.335 (2)	N1—S1	1.6752 (13)
C10—O4	1.440 (2)	O1—S1	1.4204 (13)
C10—C11	1.488 (3)	O2—S1	1.4159 (13)
			
C2—C1—C6	121.18 (16)	C10—C11—H11B	109.5
C2—C1—N1	131.69 (15)	H11A—C11—H11B	109.5
C6—C1—N1	107.11 (14)	C10—C11—H11C	109.5
C3—C2—C1	117.02 (18)	H11A—C11—H11C	109.5
C3—C2—H2	121.5	H11B—C11—H11C	109.5
C1—C2—H2	121.5	C13—C12—C17	121.55 (16)
C2—C3—C4	122.4 (2)	C13—C12—S1	119.47 (13)
C2—C3—H3	118.8	C17—C12—S1	118.95 (13)
C4—C3—H3	118.8	C12—C13—C14	118.77 (18)
C5—C4—C3	120.76 (18)	C12—C13—H13	120.6
C5—C4—H4	119.6	C14—C13—H13	120.6
C3—C4—H4	119.6	C15—C14—C13	120.20 (19)
C4—C5—C6	119.07 (19)	C15—C14—H14	119.9
C4—C5—H5	120.5	C13—C14—H14	119.9
C6—C5—H5	120.5	C14—C15—C16	120.64 (18)
C5—C6—C1	119.62 (18)	C14—C15—H15	119.7
C5—C6—C7	132.45 (17)	C16—C15—H15	119.7
C1—C6—C7	107.92 (14)	C15—C16—C17	120.23 (18)
C8—C7—C6	108.45 (15)	C15—C16—H16	119.9
C8—C7—H7	125.8	C17—C16—H16	119.9
C6—C7—H7	125.8	C16—C17—C12	118.60 (18)
C7—C8—N1	109.27 (14)	C16—C17—H17	120.7
C7—C8—C9	125.71 (15)	C12—C17—H17	120.7
N1—C8—C9	124.28 (13)	C1—N1—C8	107.24 (12)
O3—C9—O4	124.55 (16)	C1—N1—S1	125.14 (11)
O3—C9—C8	126.12 (16)	C8—N1—S1	126.64 (11)
O4—C9—C8	109.22 (14)	C9—O4—C10	116.12 (15)
O4—C10—C11	106.90 (19)	O2—S1—O1	119.66 (8)
O4—C10—H10A	110.3	O2—S1—N1	108.04 (7)
C11—C10—H10A	110.3	O1—S1—N1	105.26 (7)
O4—C10—H10B	110.3	O2—S1—C12	110.04 (8)
C11—C10—H10B	110.3	O1—S1—C12	108.23 (8)
H10A—C10—H10B	108.6	N1—S1—C12	104.50 (7)
C10—C11—H11A	109.5		
			
C6—C1—C2—C3	−0.2 (3)	C13—C12—C17—C16	−1.2 (2)
N1—C1—C2—C3	−178.06 (18)	S1—C12—C17—C16	−179.06 (14)
C1—C2—C3—C4	0.1 (3)	C2—C1—N1—C8	177.65 (17)
C2—C3—C4—C5	−0.4 (3)	C6—C1—N1—C8	−0.45 (17)
C3—C4—C5—C6	0.7 (3)	C2—C1—N1—S1	−13.1 (3)
C4—C5—C6—C1	−0.7 (3)	C6—C1—N1—S1	168.80 (11)
C4—C5—C6—C7	177.8 (2)	C7—C8—N1—C1	0.77 (18)
C2—C1—C6—C5	0.5 (3)	C9—C8—N1—C1	−169.90 (14)
N1—C1—C6—C5	178.83 (16)	C7—C8—N1—S1	−168.27 (12)
C2—C1—C6—C7	−178.36 (16)	C9—C8—N1—S1	21.1 (2)
N1—C1—C6—C7	−0.01 (18)	O3—C9—O4—C10	4.4 (2)
C5—C6—C7—C8	−178.14 (19)	C8—C9—O4—C10	−171.90 (15)
C1—C6—C7—C8	0.49 (19)	C11—C10—O4—C9	167.30 (18)
C6—C7—C8—N1	−0.78 (19)	C1—N1—S1—O2	−137.60 (13)
C6—C7—C8—C9	169.72 (15)	C8—N1—S1—O2	29.58 (15)
C7—C8—C9—O3	−153.97 (18)	C1—N1—S1—O1	−8.67 (15)
N1—C8—C9—O3	15.2 (3)	C8—N1—S1—O1	158.50 (13)
C7—C8—C9—O4	22.3 (2)	C1—N1—S1—C12	105.25 (14)
N1—C8—C9—O4	−168.58 (14)	C8—N1—S1—C12	−87.58 (14)
C17—C12—C13—C14	0.0 (2)	C13—C12—S1—O2	3.79 (15)
S1—C12—C13—C14	177.82 (13)	C17—C12—S1—O2	−178.30 (12)
C12—C13—C14—C15	1.2 (3)	C13—C12—S1—O1	−128.64 (13)
C13—C14—C15—C16	−1.2 (3)	C17—C12—S1—O1	49.27 (14)
C14—C15—C16—C17	−0.1 (3)	C13—C12—S1—N1	119.56 (13)
C15—C16—C17—C12	1.3 (3)	C17—C12—S1—N1	−62.53 (14)

### Hydrogen-bond geometry (Å, °)

**Table:**

*D*—H···*A*	*D*—H	H···*A*	*D*···*A*	*D*—H···*A*
C2—H2···O1	0.93	2.29	2.839 (2)	117.
C13—H13···O2	0.93	2.55	2.923 (2)	105.
